# 

**Published:** 1913-09-27

**Authors:** 


Mr. T. Hayhurst, M.B., Ch.B., has been appointed resident medical officer at the Manchester Children's Hospital, Pendlebury.

### Cambridge and District Workers' Hospital Fund.

THE third annual meeting of the Cambridge and District Workers' Hospital Fund was recently held at Addenbrooke's Hospital, Cambridge. Colonel T. W. Harding, LL.D., J.P., president, presided over the meeting, and there were also present: Mrs. George Street, vice-president; Mrs. Mellish Clark and Mrs. J. V. Pryor, representing the Cambridge and District Nursing Association; Mr. F. Cant, hon. treasurer; Mr. E. J. Papworth, hon. secretary; Miss M. G. Montgomery, matron; and Mr. Richard J. Coles, secretary-superintendent of Addenbrooke's, together with a large attendance of delegates from the various centres.

In accordance with the rules seven-tenths of the net receipts, £380, was allocated to Addenbrooke's Hospital, and in addition a further sum of £40 was specially voted, making a total of £420 for Addenbrooke's, a cheque for which was handed to Colonel Harding, chairman of the finance committee; £70 was voted to the Cambridge and District Nurses' Association, £10 to Cherry Hinton District Nurses' Association, £5 5s. to the Surgical Aid Society, and an honorarium of £7 to the Secretary, Mr. E. J. Papworth. The balance was transferred to the convalescent branch of the Fund, which has been of great service, having during the past year contributed £16 16s. to enable twelve patients each to spend three weeks at a convalescent home, thus restoring them to a state of convalescence.

The Workers' Fund, writes our correspondent, which in its infancy was looked upon with some suspicion and certainly great speculation as to its possibility of success, has come to stay, and although, owing to the trying circumstances of the past year largely due to the coming into operation of the Insurance Act, the amount collected is considerably less than last, there is every reason to hope and believe that with renewed zeal and efforts in the course of a few years the workers will raise the handsome sum of £1,000 a year and so accomplish the noble task which they set out to do.

During the three years the Workers' Fund has been in existence the workers have thereby contributed no less a sum than £1,808 for the benefit of the sick and suffering, and of this £1,300 has been given to Addenbrooke's.

Before the meeting of the Workers' Fund closed the executive committee and delegates unanimously expressed great regret at the departure of Miss Montgomery from Addenbrooke's, and offered her their sincere wishes for success and happiness in the more important post to which she had been called. The officers of the Fund for the ensuing year were duly elected.

### AN INFIRMARY FUNCTION AT BIRMINGHAM.

AN interesting ceremony took place recently at the Selly Oak Infirmary, Birmingham, when a presentation was made to Dr. Ellis, late resident medical officer of that institution. The presentation, which took the form of an antique grandfather's clock, was made by Dr. Hollinshead (visiting medical officer), who is the oldest officer of the late King's Norton Union. The occasion was Dr. Ellis's departure from the infirmary to take up the duties of his appointment as chief medical officer of the Dudley Road Infirmary, Birmingham. Dr. Hollinshead asked Dr. Ellis to accept the gift as a mark of the high regard in which he is held by all who had been associated with him during the four years of his tenure of office. It was a matter of regret that all the subscribers were not able to be present to meet Dr. and Mrs. Ellis, as every past member of the staff was identified with the presentation.

### A WORKPEOPLE'S HOSPITAL "AT HOME."

On the invitation of the Chairman and Committee of the Ancoats Hospital, Manchester, about 170 friends and supporters of the Workpeople's Fund Committee had an enjoyable "At Home" at the hospital last Saturday. The band of the Ardwick Industrial School was in attendance. During the afternoon the friends were conducted round the wards, and were greatly interested in all they saw. The children's wards and *x*-ray rooms came in for a large amount of attention, the interest taken in these departments being most marked. Tea was provided in the large out-patient waiting-hall, which had been artistically arranged by the Matron and her staff. The Chairman of the Committee, Mr. W. Armitage, the Honorary Secretary, Mr. Gaddum, Councillor Grimes, and others addressed the assembly, after which dancing was indulged in. Among those present were Councillors J. C. Grimes and Pendlebury, Mr. W. Armitage (chairman of the committee of management), Mr. A. E. Gaddum (honorary secretary), Mr. J. Hawkes (chairman of the Workpeople's Fund Committee), and Mr. C. B. Cross (honorary secretary).

